# Factors that influence outcomes and device use for pediatric cochlear implant recipients with unilateral hearing loss

**DOI:** 10.3389/fnhum.2023.1141065

**Published:** 2023-05-12

**Authors:** Lisa R. Park, Erika B. Gagnon, Margaret T. Dillon

**Affiliations:** Department of Otolaryngology/Head and Neck Surgery, The University of North Carolina, Chapel Hill, Chapel Hill, NC, United States

**Keywords:** SSD, UHL, single-sided deafness, outcomes, HHP, wear time

## Abstract

**Introduction:**

Candidacy criteria for cochlear implantation in the United States has expanded to include children with single-sided deafness (SSD) who are at least 5 years of age. Pediatric cochlear implant (CI) users with SSD experience improved speech recognition with increased daily device use. There are few studies that report the hearing hour percentage (HHP) or the incidence of non-use for pediatric CI recipients with SSD. The aim of this study was to investigate factors that impact outcomes in children with SSD who use CIs. A secondary aim was to identify factors that impact daily device use in this population.

**Methods:**

A clinical database query revealed 97 pediatric CI recipients with SSD who underwent implantation between 2014 and 2022 and had records of datalogs. The clinical test battery included speech recognition assessment for CNC words with the CI-alone and BKB-SIN with the CI plus the normal-hearing ear (combined condition). The target and masker for the BKB-SIN were presented in collocated and spatially separated conditions to evaluate spatial release from masking (SRM). Linear mixed-effects models evaluated the influence of time since activation, duration of deafness, HHP, and age at activation on performance (CNC and SRM). A separate linear mixed-effects model evaluated the main effects of age at testing, time since activation, duration of deafness, and onset of deafness (stable, progressive, or sudden) on HHP.

**Results:**

Longer time since activation, shorter duration of deafness, and higher HHP were significantly correlated with better CNC word scores. Younger age at device activation was not found to be a significant predictor of CNC outcomes. There was a significant relationship between HHP and SRM, with children who had higher HHP experiencing greater SRM. There was a significant negative correlation between time since activation and age at test with HHP. Children with sudden hearing loss had a higher HHP than children with progressive and congenital hearing losses.

**Conclusion:**

The present data presented here do not support a cut-off age or duration of deafness for pediatric cochlear implantation in cases of SSD. Instead, they expand on our understanding of the benefits of CI use in this population by reviewing the factors that influence outcomes in this growing patient population. Higher HHP, or greater percentage of time spent each day using bilateral input, was associated with better outcomes in the CI-alone and in the combined condition. Younger children and those within the first months of use had higher HHP. Clinicians should discuss these factors and how they may influence CI outcomes with potential candidates with SSD and their families. Ongoing work is investigating the long-term outcomes in this patient population, including whether increasing HHP after a period of limited CI use results in improved outcomes.

## Introduction

Candidacy criteria for cochlear implantation in the United States has expanded to include children aged 5 years and older with severe-to-profound unilateral hearing loss (UHL), also known as single-sided deafness (SSD). As the procedure becomes more common, research turns toward optimizing outcomes and predicting benefit with cochlear implant (CI) use. These are important factors for identifying appropriate candidates, providing appropriate counseling, and supporting optimal performance with the CI.

There is a great deal of variability when it comes to CI outcomes for pediatric recipients. For that reason, factors that influence auditory and spoken language outcomes among children with bilateral hearing loss have been studied extensively. Factors typically associated with better CI outcomes include bilateral as opposed to unilateral implantation (Sharma et al., 2020; Eskridge et al., 2021), better pre-operative hearing performance (Niparko et al., 2010), quality parent-child interactions (Niparko et al., 2010; Duchesne and Marschark, 2019), favorable social determinants of health (Niparko et al., 2010; Duchesne and Marschark, 2019; Sharma et al., 2020), fewer comorbidities (Duchesne and Marschark, 2019; Sharma et al., 2020), and etiology (Black et al., 2011). Importantly, previous investigations of these factors were specific to children with hearing loss in both ears. It is unclear whether the same patterns would be observed for children with SSD and a CI.

Some factors that are most consistently observed to influence auditory and spoken language outcomes in children with bilateral hearing loss and could potentially impact CI outcomes in cases of SSD are related to time. Younger age at implantation is often cited as an important factor, with those implanted at younger ages achieving better outcomes in language, articulation, and speech recognition (Niparko et al., 2010; Black et al., 2011; Leigh et al., 2013, 2016; Tobey et al., 2013; Miyamoto et al., 2017; Duchesne and Marschark, 2019; Sharma et al., 2020; Chweya et al., 2021; Dettman et al., 2021; Culbertson et al., 2022). Performance has been found to increase with CI use over time (Green et al., 2005). Longer durations of deafness prior to implantation have been found to adversely impact speech recognition (Shea et al., 1990; Green et al., 2005; Park et al., 2021a). With the advent of datalogging, daily device use has emerged as a significant predictor of language and speech recognition (Easwar et al., 2018; Wiseman and Warner-Czyz, 2018; Park et al., 2019; Gagnon et al., 2021); longer daily wear time is associated with better performance. These time factors–age, time since activation, duration of deafness, and time wearing the device–are important to include in studies investigating CI outcomes.

There is discrepancy in the field as to whether cochlear implantation should be recommended for children with SSD who have longer duration of hearing loss. Some suggest restricting implantation in cases of SSD to less than 4 years duration of deafness, likely due to what is known about the development of audition in children with bilateral hearing loss (Rauch et al., 2021; Cushing et al., 2022). However, there is little evidence for these cut-offs in the literature (Cohen and Svirsky, 2019; Kurz et al., 2019; Benchetrit et al., 2021; Nassiri et al., 2022). Binaural hearing abilities continue to improve as children age into elementary school (Vaillancourt et al., 2008; Van Deun et al., 2010; Yuen and Yuan, 2014; Flanagan et al., 2021; Kane et al., 2021). Additionally, maturation of the auditory system has been observed into the teen years (Litovsky, 2015; Corbin et al., 2016). Studies of children with SSD who use CI allude to duration of deafness impacting outcomes, however, duration of daily device use was not included in their analyses (Arndt et al., 2015; Távora-Vieira and Rajan, 2015; Sladen et al., 2017; Zeitler et al., 2019; Rauch et al., 2021). In addition, many of these studies involve fewer than 15 participants with heterogeneous characteristics and varied test batteries. Age at implantation and time since activation are rarely used as a continuous variable in these studies, instead, participants are grouped by ranges of time, such as small groups of 3–4 children implanted between ages 1–3 years, 4–5 years, and over age 5 years (Rauch et al., 2021).

Daily device use was found to encompass 60% of the variability in language outcomes for children with bilateral hearing loss who use CIs (Park et al., 2019), yet it is rarely included as a dependent variable in studies of children with SSD who use a CI. While many studies report daily device use (Arndt et al., 2015; Távora-Vieira and Rajan, 2015; Zeitler et al., 2019; Ehrmann-Mueller et al., 2020; Ganek et al., 2020; Deep et al., 2021; Rauch et al., 2021), few include the amount of time the child listened with the device as a dependent variable. In a study of cortical lateralization in 22 children with SSD using a CI, Lee et al. (2020) found that greater daily device use in children with early onset SSD (e.g., prior to age 4 years) was associated with a change toward more typical lateralization in the brain from the CI ear. For those with later onset of deafness (e.g., after age 12 years), CI use was on average 3.8 h shorter than in the early onset group. Device use in general helped to keep the normal hearing ear from showing abnormal cortical responses, but limited use did not improve deterioration of the auditory pathways in the CI ear. The strengthening of auditory pathways continued over time for the early-onset group as well, however, the authors did not note the effects of age at test, age at onset, or duration of deafness.

Daily device use has been shown to influence CI performance in children with bilateral hearing loss (Easwar et al., 2018; Wiseman and Warner-Czyz, 2018; Park et al., 2019; Gagnon et al., 2021). The patterns of daily device use may differ for children with SSD than for children with bilateral hearing loss since children with SSD have the input from the normal-hearing ear to compare to the input from the CI. Children with SSD may perceive a lack of benefit or find the electric signal to be bothersome as compared to their normal-hearing ear, resulting in a lack of daily device.

The Hearing Hours Percentage metric (HHP) was developed in 2019 as a method to contextualize the amount of time CI users have access to sound as compared to their typically hearing peers (Park et al., 2019). It is calculated by finding the average amount of sleep for a typically developing child by age (Galland et al., 2012) and subtracting it from 24 h to establish the number of hours a child of a certain age is awake and hence has access to sound. The hours of device use is divided by the average awake time to calculate the HHP, the percentage of typical awake time that a child using a CI has access to sound. The influence of HHP for pediatric CI users with SSD remains unknown. In the case of SSD, the HHP would provide information about what percentage of the day is devoted to bilateral as opposed to unilateral hearing.

At our center, candidacy for cochlear implantation is considered for each ear individually. Therefore, children with various hearing loss configurations and threshold levels who are unable to benefit from acoustic amplification are considered for implantation. Since these children do not always have a profound hearing loss associated with deafness, we refer to these children as having unilateral hearing loss (UHL). Children with UHL are unique in that they have already established networks for spoken language development; thus one of the goals of CI use is to rehabilitate binaural hearing. It stands to reason that the amount of time they spend with bilateral input would be a significant factor impacting outcomes. The primary aim of the current study is to investigate time-related factors that may affect word recognition in the CI ear alone and spatial release from masking in children with UHL who receive a CI (UHL + CI; time since activation, duration of deafness, HHP, and age at activation). The secondary aim is to define time-related factors that impact daily device use in this population (age at testing, time since activation, duration of deafness, and the onset of deafness).

## Materials and methods

### Data acquisition

The clinical database was queried to identify children with UHL who received a CI before 18 years of age. Queried data included onset of moderate-to-profound UHL (congenital, progressive, or sudden), duration of UHL prior to implantation, age at CI activation, HHP at each post-activation visit, age at each visit, and all speech recognition data from monaural (CI-alone) and bilateral (CI plus NH-ear) listening conditions.

The test battery at the study site for pediatric CI recipients with UHL includes measures of speech recognition with the CI-alone and in the combined listening condition, as recommended by the American Cochlear Implant Alliance task force and endorsed by the American Academy of Audiology (Park et al., 2022). Briefly, speech recognition with the CI-alone is assessed with a recorded 50-word CNC list (Peterson and Lehiste, 1962). The materials are presented via direct audio input (DAI) and the children are allowed to adjust the input volume to a comfortable level while listening to practice stimuli. This method has been found to provide valid results (Sevier et al., 2019), particularly when compared to using contralateral masking in children with UHL (Park et al., 2021b) and has been used in other pediatric UHL + CI studies (Deep et al., 2021). Performance is scored as the percentage of words repeated correctly. Speech recognition in the combined condition (CI plus the contralateral normal-hearing ear) is evaluated with a spatial hearing task in a sound booth using the recorded BKB-SIN test materials (Bench et al., 1979) presented at 60 dBA. Performance is evaluated in two target-to-masker configurations: (1) speech and masker collocated from the front speaker (0° azimuth) and (2) speech from the front and masker directed 90° toward the NH-ear. This condition poses a challenge for patients with UHL. Performance is scored as the dB SNR-50 where the listener recognizes approximately 50% of the target speech. For the present study, benefit was evaluated as the spatial release from masking (SRM), which is the improvement in speech recognition when the masker is offset from the target speech. SRM was calculated by subtracting the score obtained in the spatially separated condition from the score obtained in the collocated condition.

### Data analysis

Two linear mixed-effects models evaluated time-related effects on performance in children with UHL + CI. One model evaluated the effects on CNC word scores, transformed to RAU, and the other on SRM. Both models included time since activation, duration of deafness, HHP, and age at activation as potential predictors. Two children were excluded from the speech recognition analyses due to outlier status. They were the only children in the sample who were implanted under 3 years of age–both undergoing implantation before 12 months of age due to meningitis. Each participant had multiple points included in the analysis since data were obtained from multiple post-activation visits, yielding a total of 198 data points from 50 subjects for the CNC analysis and 97 data points from 50 subjects for the SRM analysis. Subject was included as a random factor to account for this.

A linear mixed-effects model evaluated the influence of age at testing, time since activation, duration of deafness, and the onset of deafness on HHP. Similar to the speech recognition analyses, each participant had multiple points included in the analysis. There were a total of 562 data points from 97 subjects. R statistical software (R Core Team, 2021) was used with the subject as a random factor. Time since activation and duration of deafness were Log2 transformed for all analyses due to violations of normality. Age at activation and was also Log2 transformed in the speech recognition analysis to address normality violations. All analyses allowed for the evaluation of each individual variable while controlling for the other variables in that model.

## Results

### Factors that influence performance

Descriptive data are listed in Table 1. The database query identified 97 patients who underwent cochlear implantation at the study site between 2014 and 2022. The median time since activation at the point of data collection was 6 months for the full dataset (range = 2 weeks–6.7 years). Age at activation ranged from 6 months to 17.7 years (Median = 5.9 years) and duration of deafness ranged from 2 months to 14.0 years (Median = 3.7 years). Children implanted under 5 years of age and children with greater than 10 years of deafness were implanted off-label.

**TABLE 1:** Participant demographics.

	N (% of total) full dataset	N (% of total) CNC group	N (% of total) SRM group
Etiology			
Unknown	54 (56%)	28 (56%)	30 (60%)
Malformation	14 (14%)	6 (12%)	6 (12%)
cCMV	12 (12%)	3 (6%)	3 (6%)
Trauma	6 (6%)	6 (12%)	3 (6%)
Meningitis	5 (5%)	2 (4%)	3 (6%)
Infection	3 (3%)	3 (6%)	2 (4%)
Suspected hereditary	2 (2%)	1 (2%)	2 (4%)
Waardenburg syndrome	1 (1%)	1 (2%)	1 (2%)
Manufacturer			
MED-EL	74 (76%)	43 (86%)	40 (80%)
Cochlear	22 (24%)	7 (14%)	10 (20%)
Race/Ethnicity			
White	56 (58%)	30 (60%)	27 (54%)
African American	16 (16%)	7 (14%)	7 (14%)
Hispanic	12 (12%)	6 (12%)	6 (12%)
Mixed race	9 (9%)	5 (10%)	7 (14%)
Asian	2 (2%)	1 (2%)	2 (4%)
Native American	2 (2%)	1 (2%)	1 (2%)
Sex			
Female	50 (52%)	22 (44%)	24 (48%)
Male	47 (48%)	28 (56%)	26 (52%)
Onset of deafness			
Congenital	53 (55%)	28 (56%)	22 (44%)
Progressive	29 (30%)	13 (26%)	13 (26%)
Sudden	15 (15%)	9 (18%)	15 (30%)
NBHS results			
Pass	49 (51%)	34 (68%)	26 (52%)
Failed	38 (39%)	8 (16%)	18 (36%)
Fail, pass on rescreen	9 (9%)	7 (14%)	5 (10%)
Not screened	1 (1%)	1 (2%)	1 (2%)
Affected ear			
Left	51 (53%)	26 (52%)	26 (52%)
Right	46 (47%)	24 (48%)	24 (48%)

**FIGURE 1:**
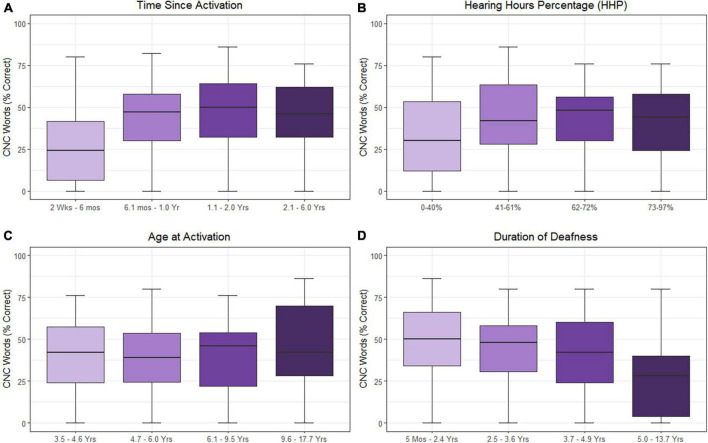
Effects of time since activation [**(A)**; *p* < 0.001], Hearing Hours Percentage [HHP; **(B)**; *p* < 0.001], age at activation [**(C)**; *p* = 0.052], and duration of deafness [**(D)**; *p* = 0.003] on CNC word scores. All variables were treated as continuous variables in the analysis but are separated into categories by quartiles for illustration. CNC words were converted to RAU prior to analysis. Duration of deafness, age at activation, and time since activation were Log_2_ transformed prior to analysis. All are shown here as with traditional units.

The results of the linear mixed-effects model assessing the factors that influence CNC word scores with the CI alone are listed in Table 2 and plotted in Figure 1 using standard percent correct units. Estimated correlations of fixed effects are available in Supplementary Table 1. Children in this model ranged from 3.8 to 17.9 years of age (*M* = 8.3 years, SD = 1.1). The mean CNC word score obtained in the CI ear alone was 39.6% (SD = 23.4). A significant positive relationship with CNC words was observed for both time since activation (Median = 1.0 year, IQR = 0.5–1.9, *p* < 0.001) and HHP (*M* = 55.4%, SD = 23.2, *p* = 0.003). Age of activation was not a significant predictor of CNC word scores (*p* = 0.052). There was a significant negative correlation between the duration of deafness (Median = 3.6 years, IQR = 2.4–4.9) and CNC words scores (*p* = 0.035). Together, these results indicate that better CNC word scores are observed for pediatric CI users with UHL who have shorter durations of deafness prior to implantation, a longer time period since activation, and a higher HHP. Age at activation did not significantly predict word recognition in this cohort.

**TABLE 2:** Results of the linear mixed-effects model investigating factors that influence word recognition in the cochlear implant (CI) ear alone.

Predictors	Estimate	SE	*t*-value	*p*-value
(Intercept)	-3.31	17.600	-0.188	0.851
Time since activation_(Log2)_	10.56	1.858	5.686	<**0**.**001**
HHP	0.51	0.101	5.020	<**0**.**001**
Age at activation_(Log2)_	9.33	4.654	2.006	0.052
Duration of deafness_(Log2)_	-7.78	2.437	-3.193	**0**.**002**
N _SubjID_	50			
Observations	198			
Marginal *R*^2^	0.280	Conditional *R*^2^		0.576

Values in bold indicate significant findings.

**FIGURE 2:**
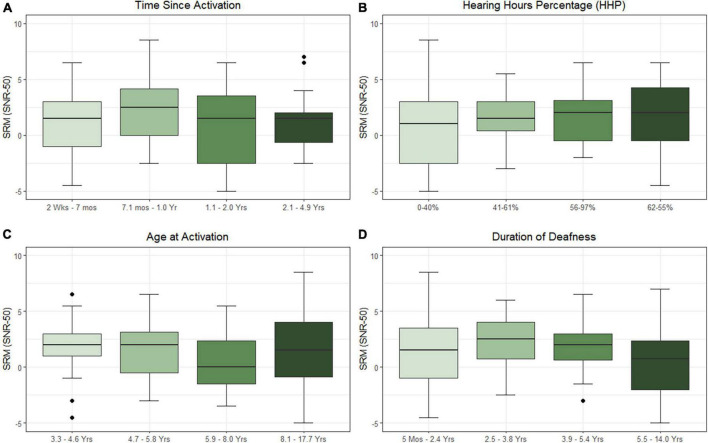
Effects of time since activation [**(A)**; *p* = 0.639], Hearing Hours Percentage [HHP; **(B)**; *p* = 0.043], age at activation [**(C)**; *p* = 0.767], and duration of deafness [**(D)**; *p* = 0.795] on spatial release from masking (SRM) when the masker is directed toward the better hearing ear. All variables were treated as continuous variables in the analysis but are separated into categories by quartiles for illustration. Duration of deafness, age at activation, and time since activation were Log_2_ transformed prior to analysis. All are shown here with traditional units.

Results of the linear mixed-effects model assessing the influence of the time-related factors (i.e., time since activation, duration of deafness, age at activation, and HHP) on SRM are listed in Table 3 and plotted in Figure 2. Estimated correlations of fixed effects are available in Supplementary Table 2. Children in this model ranged from 4.1 to 17.9 years of age (*M* = 8.0 years, SD = 3.2. The only factor reaching significance in this model was HHP (*M* = 54.9%, SD = 23.7, *p* = 0.043). Time since activation (Median = 1.0 year, IQR = 0.7–2.0, *p* = 0.392), duration of deafness (Median = 3.8 years, IQR = 0.5–5.4, *p* = 0.795), and age at activation (Median = 5.8 years, IQR = 4.6–8.0, *p* = 0.767) did not have a significant influence on children with UHL + CI.

**TABLE 3:** Results of the linear mixed-effects model investigating factors that influence sentence recognition in spatially separated noise.

Predictors	Estimate	SE	*t*-value	*p*-value
(Intercept)	-1.35	2.865	-0.472	0.639
Time since activation_(Log2)_	0.26	0.308	0.861	0.392
HHP	0.04	0.019	2.057	**0.043**
Age at activation_(Log2)_	0.23	0.769	0.298	0.767
Duration of deafness_(Log2)_	0.10	0.392	0.262	0.795
N _SubjID_	50			
Observations	97			
Marginal *R*^2^	0.061	Conditional *R*^2^		0.463

The amount of spatial release from masking when the masker was directed to the better hearing ear was used as the dependent variable. Values in bold indicate significant findings.

### Factors that influence device use

**FIGURE 3:**
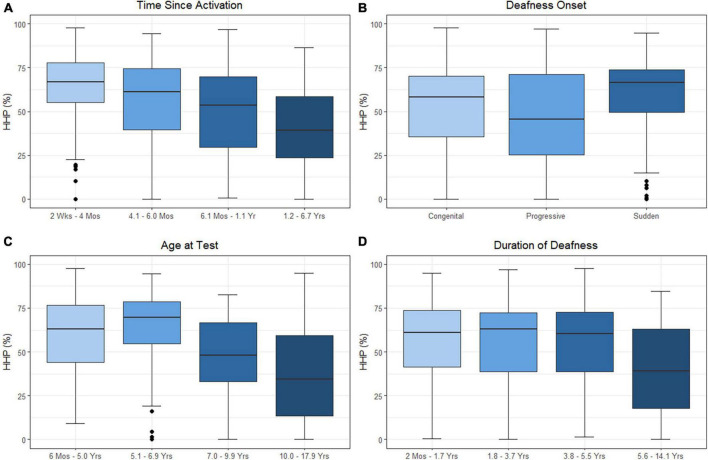
Effects of time since activation [**(A)**; *p* < 0.001], onset of deafness [**(B)**; *p* = 0.001], age at test [**(C)**; *p* < 0.001], and duration of deafness [**(D)**; *p* = 0.577] on Hearing Hours Percentage (HHP). Independent variables of age, time, and duration were treated as continuous variables in the analysis but are separated into categories by quartiles for illustration. Duration of deafness and time since activation were both Log_2_ transformed prior to analysis. All are shown here as transformed data with traditional units.

As HHP was the only factor associated with outcomes in both above models, we decided to investigate the factors that may influence wear time. The results of the linear mixed-effects model assessing time since activation, duration of deafness, age at test, and onset of hearing loss on HHP are presented in Table 4 and plotted in Figure 3. Estimated correlations of fixed effects are available in Supplementary Table 3. The age at test ranged from 0.6 to 17.9 years. The mean HHP was 52.1% (SD = 23.6). There was a significant negative correlation with HHP and both time since activation (Median = 0.9 years, IQR = 0.2–1.2, *p* < 0.001) and age at test (*M* = 7.5 years, SD = 3.6, *p* < 0.001). Onset of deafness was also significantly negatively correlated with HHP *(p* = 0.001). *Post hoc* comparisons with Bonferroni corrections applied found no significant difference in device use between those with congenital (*M* = 52.9%, SD = 22.8) or progressive losses (*M* = 46.3%, SD = 27.0; *p* = 0.771). Children with sudden hearing losses (*M* = 59.4%, SD = 23.4) had a 13.1 unit higher HHP than those with progressive losses (*p* = 0.001) and a 6.5 unit higher HHP than those with congenital hearing losses (*p* = 0.010). Duration of deafness (Median = 3.7 years, IQR = 1.7–5.5) was not a significant factor in CI use when controlling for other variables (*p* = 0.577). Taken together, these findings suggest that children with UHL + CI wear their devices less as they age and as they move further from their activation day. Children with congenital and progressive deafness wear their devices less than those with a sudden onset of deafness. Duration of deafness does not appear to significantly impact daily device use.

**TABLE 4:** Results of the linear mixed-effects model investigating factors that influence Hearing Hours Percentage (HHP) in children with UHL + CI.

Predictors	Estimates	SE	*t*-value	*p*
(Intercept)	67.32	4.855	13.868	<**0**.**001**
Time since activation_(Log2)_	-3.566	0.554	-6.432	<**0**.**001**
Duration of deafness_(Log2)_	0.93	1.661	0.560	0.577
Age at test	-3.24	0.497	-6.529	<**0**.**001**
Onset of hearing loss [Progressive]	-5.02	4.35	-1.16	0.251
Onset of hearing loss [Sudden]	18.45	6.014	3.068	**0**.**003**
N _SubjID_	97			
Observations	562			
Marginal *R*^2^	0.297	Conditional *R*^2^		0.811

For the categorical variable, onset of hearing loss, congenital was the reference variable. The comparison variable is in brackets. Values in bold indicate significant findings.

## Discussion

The primary aim of this study was to evaluate time factors that influence patient performance in children with UHL + CI. In this study, better word recognition was observed for pediatric CI users with UHL with longer time since activation, more hours of daily CI use, and shorter durations of deafness. Age at implantation did not significantly predict CNC word recognition with the CI in this sample. This may be due to the variability in the onset of severe-to-profound UHL.

Interestingly, the only time factor found to be associated with the amount of SRM children with UHL + CI experienced was HHP. An HHP value gives an estimation of how much time is dedicated to bilateral versus unilateral hearing each day. Inclusion of HHP values in future studies that assess true binaural hearing abilities (e.g., binaural squelch) may help to better understand the bilateral listening experience in this patient population. A consideration of the present data set is the use of the BKB-SIN, which has been shown to have a high degree of variability in children (Holder et al., 2016). Quality of life measures may be important to include as well as recent research suggests that children with longer durations of deafness and older age at implantation report subjective benefit (Zeitler et al., 2023). There is a need for other clinically applicable measures of binaural hearing abilities that are appropriate for children.

The second aim of this study was to define factors that impact daily device use in the pediatric UHL + CI population. The mean HHP of this cohort was found to be only 52%. Previous studies of device use in children with bilateral hearing loss have historically used hours of use rather than proportion of waking hours. Conversion from mean hours of use and mean age to HHP in those studies reveals an average HHP between 55 and 60% (Easwar et al., 2018; Wiseman and Warner-Czyz, 2018; Park et al., 2019; Deep et al., 2021), not particularly higher than the HHP found in this UHL cohort. Polonenko et al. (2017) surmised that the mean 6.2 h/day device use in their study of children with SSD + CI was shorter than what is typically seen in children with bilateral hearing loss. As that was a longitudinal study it is difficult to calculate the mean age of the participants, however, the first datalog was collected at a mean of 6.25 years of age. Conversion of 6.2 h of use and 6.25 years of age results in a 43% HHP, a number quite a bit lower than in other studies, including the current work.

Duration of deafness was not found to impact HHP. Age at test and time since activation, however, were both negatively correlated with HHP. Older children wore their devices for a shorter period of waking hours and overall use decreased over time. This finding is in contrast to other studies (Polonenko et al., 2017; Ganek et al., 2020; Deep et al., 2021) that stated that older children wore their devices more and use was consistent over time. While these studies reported datalogs of a much smaller number of children than the present work, the authors also relied on hours of use rather than HHP. As children are awake more as they age, it may be that HHP decreased in those samples. The maximum duration of time since activation in the present work was 6.7 years while the maximum in previous studies was 3.7 years. Additionally, our findings controlled for other factors (i.e., age at activation, duration of deafness, age at test, time since activation, and onset of hearing loss). Therefore, a 5-year-old child with a 3-year duration of sudden hearing loss prior to implantation and who had been using a CI for 1 year would have a higher HHP (predicted to be 91.2%) than a 14-year-old with a 3-year duration of sudden hearing loss prior to implantation and 1 year of CI use (predicted to be 31.8%).

**FIGURE 4:**
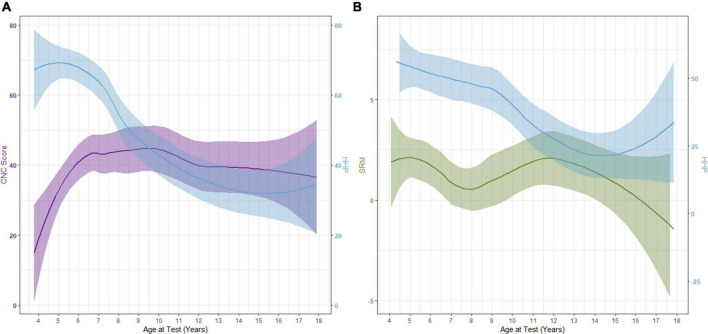
Interaction between Hearing Hours Percentage (HHP) with CNC word scores **(A)** and spatial release from masking [SRM; **(B)**] as participants age.

The interplay of daily device use and performance is complicated. Does lack of use lead to poorer outcomes, or do poorer outcomes lead to a lack of device use? As plotted in Figure 4, our data suggests that it is likely the former. In this cohort, word recognition increased while HHP decreased. Following this decrease in use, CNC word scores decrease slightly over time but are mostly stable. Similar trends are noted for SRM, however, there is a larger decrease in the amount of SRM observed after the decline in daily device use is noted. For both measures, there is a slight increase in HHP observed in the later teen years. Future research investigating long-term daily device use and outcomes will be important as these children progress to adulthood.

Like other studies, we surmise that psychosocial factors play a role in the wear time of older children (Thomas et al., 2017; Deep et al., 2021). Adolescence brings social change and challenges that can make it difficult to accept an assistive device (Borman and Schneider, 1998; Blakemore and Mills, 2014). In cases of UHL, children can communicate with only one ear and no one needs to be made aware of their disability. When they receive a CI, wearing the external device makes the disability obvious and draws attention to their differences. Interestingly, this is not unique to hearing technology. Children with Type 1 Diabetes are less compliant with intervention in their teen years (Weissberg-Benchell et al., 1995) as are teens who use ankle-foot orthotics for walking support (Wingstrand et al., 2014). These findings draw attention to the need for appropriate counseling of candidates and recipients in this age group. Studies of adult CI recipients with UHL report that non-users tend to have unrealistic expectations pre-operatively and are overwhelmed with the amount of rehabilitation recommended for optimal outcomes (Távora-Vieira et al., 2020). In this study children with UHL + CI who had a sudden onset of hearing loss used their CI for a larger portion of their day than children with congenital or progressive hearing loss. This is not surprising as they have grown accustomed to bilateral hearing while their contemporaries are accustomed to unilateral hearing.

This study provides evidence of the impact of time variables on pediatric UHL + CI outcomes and the importance of daily device use. We have been able to present a large, diverse cohort of children with UHL + CI with maximum durations of deafness as long as 14 years, a maximum of over 6 years since activation, and a wide implantation age of 6 months to 17.9 years. When considering cochlear implantation for the pediatric UHL + CI candidate, duration of deafness should be an important part of counseling. The evidence presented here, however, does not support precluding CI consideration based on age or duration of deafness. The present data suggest that increasing daily device use positively influences outcomes. This is a factor that can be manipulated and potentially shaped through counseling on realistic expectations. Pediatric patients with UHL entering their teen years, should have realistic goals, a thorough understanding of what full-time device use means, knowledge of what the device looks like, and certainty that they are willing to commit to wearing the external device before deciding to pursue a CI.

## Data availability statement

The raw data supporting the conclusions of this article will be made available by the authors, without undue reservation.

## Ethics statement

The studies involving human participants were reviewed and approved by the University of North Carolina, Chapel Hill. Written informed consent to participate in this study was provided by the participants’ legal guardian/next of kin.

## Author contributions

LP and EG contributed to the data collection. LP and MD contributed to the data analysis. All authors wrote and revised the manuscript and contributed to the article and approved the submitted version.

## References

[B1] Arndt S. Prosse S. Laszig R. Wesarg T. Aschendorff A. Hassepass F. (2015). Cochlear implantation in children with single-sided deafness: Does aetiology and duration of deafness matter? *Audiol. Neuro Otol.* 20(Suppl 1) 21–30. 10.1159/000380744 25999052

[B2] Bench J. Kowal A. Bamford J. (1979). The BKB (Bamford-Kowal-Bench) sentence lists for partially-hearing children. *Br. J. Audiol.* 13 108–112. 10.3109/03005367909078884 486816

[B3] Benchetrit L. Ronner E. A. Anne S. Cohen M. S. (2021). Cochlear implantation in children with single-sided deafness: A systematic review and meta-analysis. *JAMA Otolaryngol. Head Neck Surg.* 147 58–69. 10.1001/jamaoto.2020.3852 33151295 PMC7645748

[B4] Black J. Hickson L. Black B. Perry C. (2011). Prognostic indicators in paediatric cochlear implant surgery: A systematic literature review. *Cochlear Implants Int.* 12 67–93. 10.1179/146701010X486417 21756501

[B5] Blakemore S.-J. Mills K. L. (2014). Is adolescence a sensitive period for sociocultural processing? *Ann. Rev. Psychol.* 65 187–207. 10.1146/annurev-psych-010213-115202 24016274

[B6] Borman K. Schneider B. (1998). “Identity formation in adolescence,” in *The adolescent years: Social influences and educational challenges: Ninety-seventh yearbook of the National Society for the Study of Education, Part 1 The National Society for the Study of Education*, eds Borman K. Schneider B. (Washington, DC: APA), 18–41.

[B7] Chweya C. M. May M. M. DeJong M. D. Baas B. S. Lohse C. M. Driscoll C. L. W. (2021). Language and audiological outcomes among infants implanted before 9 and 12 months of age versus older children: A continuum of benefit associated with cochlear implantation at successively younger ages. *Otol. Neurotol.* 42 686–693. 10.1097/MAO.0000000000003011 33710159

[B8] Cohen S. M. Svirsky M. A. (2019). Duration of unilateral auditory deprivation is associated with reduced speech perception after cochlear implantation: A single-sided deafness study. *Cochlear Implants Int.* 20 51–56. 10.1080/14670100.2018.1550469 30486762 PMC6335158

[B9] Corbin N. E. Bonino A. Y. Buss E. Leibold L. J. (2016). Development of open-set word recognition in children: Speech-shaped noise and two-talker speech maskers. *Ear Hear.* 37 55–63. 10.1097/AUD.0000000000000201 26226605 PMC4684436

[B10] Culbertson S. R. Dillon M. T. Richter M. E. Brown K. D. Anderson M. R. Hancock S. L. (2022). Younger age at cochlear implant activation results in improved auditory skill development for children with congenital deafness. *J. Speech Lang. Hear. Res.* 65 3539–3547. 10.1044/2022_JSLHR-22-00039 36001854 PMC9913281

[B11] Cushing S. L. Purcell P. L. Papaiaonnou V. Neghandi J. Daien M. Blaser S. I. (2022). Hearing instability in children with congenital cytomegalovirus: Evidence and neural consequences. *Laryngoscope* 132(Suppl 11) S1–S24. 10.1002/lary.30108 35302239

[B12] Deep N. L. Gordon S. A. Shapiro W. H. Waltzman S. B. Roland J. T. Friedmann D. R. (2021). Cochlear implantation in children with single-sided deafness. *Laryngoscope* 131 E271–E277. 10.1002/lary.28561 32065422

[B13] Dettman S. Choo D. Au A. Luu A. Dowell R. (2021). Speech perception and language outcomes for infants receiving cochlear implants before or after 9 months of age: Use of category-based aggregation of data in an unselected pediatric cohort. *J. Speech Lang. Hear. Res.* 64 1023–1039. 10.1044/2020_JSLHR-20-00228 33630667

[B14] Duchesne L. Marschark M. (2019). Effects of age at cochlear implantation on vocabulary and grammar: A review of the evidence. *Am. J. Speech Lang. Pathol.* 28 1673–1691. 10.1044/2019_AJSLP-18-0161 31513745

[B15] Easwar V. Sanfilippo J. Papsin B. Gordon K. (2018). Impact of consistency in daily device use on speech perception abilities in children with cochlear implants: Datalogging evidence. *J. Am. Acad. Audiol.* 29 835–846. 10.3766/jaaa.17051 30278868

[B16] Ehrmann-Mueller D. Kurz A. Kuehn H. Rak K. Mlynski R. Hagen R. (2020). Usefulness of cochlear implantation in children with single sided deafness. *Int. J. Pediatr. Otorhinolaryngol.* 130:109808. 10.1016/j.ijporl.2019.109808 31809969

[B17] Eskridge H. R. Park L. R. Brown K. D. (2021). The impact of unilateral, simultaneous, or sequential cochlear implantation on pediatric language outcomes. *Cochlear Implants Int.* 22 187–194. 10.1080/14670100.2020.1871267 33430719

[B18] Flanagan S. A. Moore B. C. J. Wilson A. M. Gabrielczyk F. C. MacFarlane A. Mandke K. (2021). Development of binaural temporal fine structure sensitivity in children. *J. Acoust. Soc. Am.* 150:2967. 10.1121/10.0006665 34717481

[B19] Gagnon E. B. Eskridge H. Brown K. D. Park L. R. (2021). The impact of cumulative cochlear implant wear time on spoken language outcomes at age 3 years. *J. Speech Lang. Hear. Res.* 64 1369–1375. 10.1044/2020_JSLHR-20-00567 33784469 PMC8608196

[B20] Galland B. C. Taylor B. J. Elder D. E. Herbison P. (2012). Normal sleep patterns in infants and children: A systematic review of observational studies. *Sleep Med. Rev.* 16 213–222. 10.1016/j.smrv.2011.06.001 21784676

[B21] Ganek H. V. Cushing S. L. Papsin B. C. Gordon K. A. (2020). Cochlear implant use remains consistent over time in children with single-sided deafness. *Ear Hear.* 41 678–685. 10.1097/AUD.0000000000000797 31567563

[B22] Green K. M. J. Julyan P. J. Hastings D. L. Ramsden R. T. (2005). Auditory cortical activation and speech perception in cochlear implant users: Effects of implant experience and duration of deafness. *Hear. Res.* 205 184–192. 10.1016/j.heares.2005.03.016 15953527

[B23] Holder J. T. Sheffield S. W. Gifford R. H. (2016). Speech understanding in children with normal hearing: Sound field normative data for BabyBio, BKB-SIN, and QuickSIN. *Otol. Neurotol.* 37:e50–55. 10.1097/MAO.0000000000000907 26756155

[B24] Kane S. G. Buss E. Grose J. H. (2021). Binaural frequency modulation detection in school-age children. young adults older adults: Effects of interaural modulator phase. *Ear Hear.* 42 691–699. 10.1097/AUD.0000000000000975 33306546 PMC8087618

[B25] Kurz A. Grubenbecher M. Rak K. Hagen R. Kühn H. (2019). The impact of etiology and duration of deafness on speech perception outcomes in SSD patients. *Eur. Arch. Oto Rhino Laryngol.* 276 3317–3325. 10.1007/s00405-019-05644-w 31535291

[B26] Lee H.-J. Smieja D. Polonenko M. J. Cushing S. L. Papsin B. C. Gordon K. A. (2020). Consistent and chronic cochlear implant use partially reverses cortical effects of single sided deafness in children. *Sci. Rep.* 10:21526. 10.1038/s41598-020-78371-6 33298987 PMC7726152

[B27] Leigh J. R. Dettman S. J. Dowell R. C. (2016). Evidence-based guidelines for recommending cochlear implantation for young children: Audiological criteria and optimizing age at implantation. *Int. J. Audiol.* 55(Suppl 2) S9–S18. 10.3109/14992027.2016.1157268 27142630

[B28] Leigh J. Dettman S. Dowell R. Briggs R. (2013). Communication development in children who receive a cochlear implant by 12 months of age. *Otol. Neurotol.* 34 443–450. 10.1097/MAO.0b013e3182814d2c 23442570

[B29] Litovsky R. (2015). Development of the auditory system. *Handb. Clin. Neurol.* 129 55–72. 10.1016/B978-0-444-62630-1.00003-2 25726262 PMC4612629

[B30] Miyamoto R. T. Colson B. Henning S. Pisoni D. (2017). Cochlear implantation in infants below 12 months of age. *World J. Otorhinolaryngol. Head Neck Surg.* 3 214–218. 10.1016/j.wjorl.2017.12.001 29780965 PMC5956135

[B31] Nassiri A. M. Wallerius K. P. Saoji A. A. Neff B. A. Driscoll C. L. W. Carlson M. L. (2022). Impact of duration of deafness on speech perception in single-sided deafness cochlear implantation in adults. *Otol. Neurotol.* 43 e45–e49. 10.1097/MAO.0000000000003357 34889841

[B32] Niparko J. K. Tobey E. A. Thal D. J. Eisenberg L. S. Wang N.-Y. Quittner A. L. (2010). Spoken language development in children following cochlear implantation. *J. Am. Med. Assoc.* 303 1498–1506. 10.1001/jama.2010.451 20407059 PMC3073449

[B33] Park L. R. Gagnon E. B. Thompson E. Brown K. D. (2019). Age at full-time use predicts language outcomes better than age of surgery in children who use cochlear implants. *Am. J. Audiol.* 28 986–992. 10.1044/2019_AJA-19-0073 31721595

[B34] Park L. R. Griffin A. M. Sladen D. P. Neumann S. Young N. M. (2022). American cochlear implant alliance task force guidelines for clinical assessment and management of cochlear implantation in children with single-sided deafness. *Ear Hear.* 43 255–267. 10.1097/AUD.0000000000001204 35213890 PMC8862768

[B35] Park L. R. Perkins E. L. Woodard J. S. Brown K. D. (2021a). Delaying cochlear implantation impacts postoperative speech perception of nontraditional pediatric candidates. *Audiol. Neuro Otol.* 26 182–187. 10.1159/000510693 33352551

[B36] Park L. R. Preston E. Noxon A. S. Dillon M. T. (2021b). Comparison of test methods to assess the implanted ear alone for pediatric cochlear implant recipients with single-sided deafness. *Cochlear Implants Int.* 22 283–290. 10.1080/14670100.2021.1903715 33761831

[B37] Peterson G. E. Lehiste I. (1962). Revised CNC lists for auditory tests. *J. Speech Hear. Disord.* 27 62–70. 10.1044/jshd.2701.62 14485785

[B38] Polonenko M. J. Papsin B. C. Gordon K. A. (2017). Children with single-sided deafness use their cochlear implant. *Ear Hear.* 38 681–689. 10.1097/AUD.0000000000000452 28542017

[B39] R Core Team (2021). *R: A language and environment for statistical computing (4.0.2) [Computer software].* Vienna: R Foundation for Statistical Computing.

[B40] Rauch A.-K. Arndt S. Aschendorff A. Beck R. Speck I. Ketterer M. C. (2021). Long-term results of cochlear implantation in children with congenital single-sided deafness. *Eur. Arch. Oto Rhino Laryngol.* 278 3245–3255. 10.1007/s00405-020-06409-6 33079248 PMC8328851

[B41] Sevier J. D. Choi S. Hughes M. L. (2019). Use of direct-connect for remote speech-perception testing in cochlear implants. *Ear Hear.* 40 1162–1173. 10.1097/AUD.0000000000000693 30640730 PMC9165643

[B42] Sharma S. D. Cushing S. L. Papsin B. C. Gordon K. A. (2020). Hearing and speech benefits of cochlear implantation in children: A review of the literature. *Int. J. Pediatr. Otorhinolaryngol.* 133:109984. 10.1016/j.ijporl.2020.109984 32203759

[B43] Shea J. J. Domico E. H. Orchik D. J. (1990). Speech recognition ability as a function of duration of deafness in multichannel cochlear implant patients. *Laryngoscope* 100 223–226. 10.1288/00005537-199003000-00002 2308444

[B44] Sladen D. P. Frisch C. D. Carlson M. L. Driscoll C. L. W. Torres J. H. Zeitler D. M. (2017). Cochlear implantation for single-sided deafness: A multicenter study. *Laryngoscope* 127 223–228. 10.1002/lary.26102 27346874

[B45] Távora-Vieira D. Acharya A. Rajan G. P. (2020). What can we learn from adult cochlear implant recipients with single-sided deafness who became elective non-users? *Cochlear Implants Int.* 21 220–227. 10.1080/14670100.2020.1733746 32122282

[B46] Távora-Vieira D. Rajan G. P. (2015). Cochlear implantation in children with congenital and noncongenital unilateral deafness: A case series. *Otol. Neurotol.* 36 235–239. 10.1097/MAO.0000000000000677 25415465

[B47] Thomas J. P. Neumann K. Dazert S. Voelter C. (2017). Cochlear implantation in children with congenital single-sided deafness. *Otol. Neurotol.* 38 496–503. 10.1097/MAO.0000000000001343 28288475

[B48] Tobey E. A. Thal D. Niparko J. K. Eisenberg L. S. Quittner A. L. Wang N.-Y. (2013). Influence of implantation age on school-age language performance in pediatric cochlear implant users. *Int. J. Audiol.* 52 219–229. 10.3109/14992027.2012.759666 23448124 PMC3742378

[B49] Vaillancourt V. Laroche C. Giguère C. Soli S. D. (2008). Establishment of age-specific normative data for the canadian French version of the hearing in noise test for children. *Ear Hear.* 29 453–466. 10.1097/01.aud.0000310792.55221.0c 18349705

[B50] Van Deun L. van Wieringen A. Wouters J. (2010). Spatial speech perception benefits in young children with normal hearing and cochlear implants. *Ear Hear.* 31 702–713. 10.1097/AUD.0b013e3181e40dfe 20548238

[B51] Weissberg-Benchell J. Glasgow A. M. Tynan W. D. Wirtz P. Turek J. Ward J. (1995). Adolescent diabetes management and mismanagement. *Diabetes Care* 18 77–82. 10.2337/diacare.18.1.77 7698052

[B52] Wingstrand M. Hägglund G. Rodby-Bousquet E. (2014). Ankle-foot orthoses in children with cerebral palsy: A cross sectional population based study of 2200 children. *BMC Musculosk. Disord.* 15:327. 10.1186/1471-2474-15-327 25274143 PMC4192348

[B53] Wiseman K. B. Warner-Czyz A. D. (2018). Inconsistent device use in pediatric cochlear implant users: Prevalence and risk factors. *Cochlear Implants Int.* 19 131–141. 10.1080/14670100.2017.1418161 29299970

[B54] Yuen K. C. P. Yuan M. (2014). Development of spatial release from masking in mandarin-speaking children with normal hearing. *J. Speech Language Hear. Res.* 57 2005–2023. 10.1044/2014_JSLHR-H-13-0060 24950448

[B55] Zeitler D. M. Dunn C. Schwartz S. R. McCoy J. L. Jamis C. Chi D. H. (2023). Health-related quality of life in children with unilateral sensorineural hearing loss following cochlear implantation. *Otolaryngol. Head Neck Surg.* (in press). 10.1002/ohn.165 36934432 PMC10213080

[B56] Zeitler D. M. Sladen D. P. DeJong M. D. Torres J. H. Dorman M. F. Carlson M. L. (2019). Cochlear implantation for single-sided deafness in children and adolescents. *Int. J. Pediatr. Otorhinolaryngol.* 118 128–133. 10.1016/j.ijporl.2018.12.037 30623849

