# Supplementary material for: Factors that influence outcomes and device use for pediatric cochlear implant recipients with unilateral hearing loss
Source: Front Hum Neurosci. 2023 May 12;17:1141065. doi: 10.3389/fnhum.2023.1141065 (PMC10213360; doi:10.3389/fnhum.2023.1141065)
Supplement: Supplementary file 1 [file Data_Sheet_1.docx]

Supplementary Material

Factors that Influence Outcomes and Device use for Pediatric Cochlear Implant Recipients with Unilateral Hearing Loss

# Lisa R Park, Erika B Gagnon, Margaret T Dillon ^1^

# ^1^ The University of North Carolina at Chapel Hill, Department of Otolaryngology/Head and Neck Surgery, Chapel Hill, NC, USA

# * Correspondence: Lisa R Park Lisa_Park@med.unc.edu

**
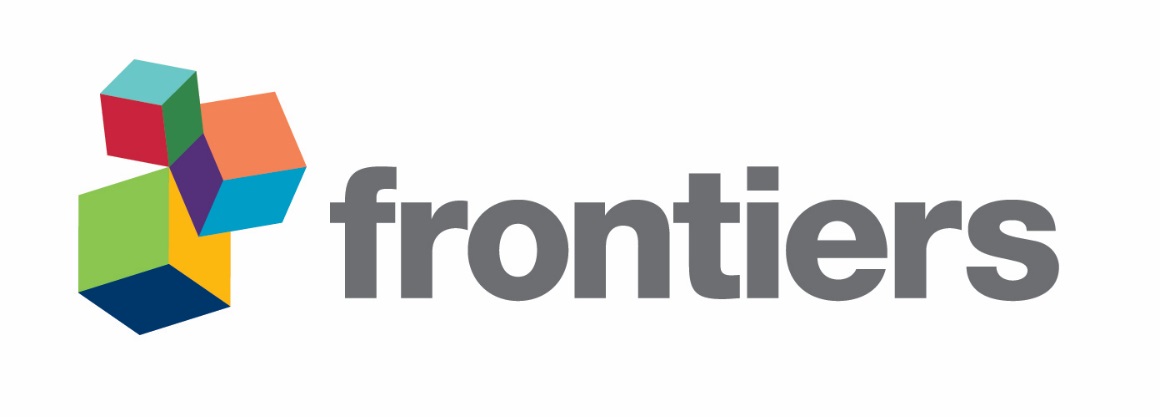
**

## Supplementary Table 1.

Estimated correlations of fixed effects derived from the linear mixed-effects model investigating factors that influence word recognition in the cochlear implant (CI) ear alone.

|  | *(Intr)* | Time since activation_(Log2)_ | HHP | Duration of deafness_(Log2)_ |
| --- | --- | --- | --- | --- |
| Time since activation_(Log2)_ | -0.376 |  |  |  |
| HHP | -0.708 | 0.440 |  |  |
| Duration of deafness_(Log2)_ | -0.923 | 0.347 | 0.505 |  |
| Age at activation_(Log2)_ | -0.421 | 0.027 | 0.191 | 0.188 |

## Supplementary Table 2.

Estimated correlations of fixed effects derived from the linear mixed-effects model investigating factors that influence sentence recognition in spatially separated noise. The amount of spatial release from masking when the masker was directed to the better-hearing ear was used as the dependent variable.

|  | *(Intr)* | Time since activation_(Log2)_ | HHP | Duration of deafness_(Log2)_ |
| --- | --- | --- | --- | --- |
| Time since activation_(Log2)_ | -0.370 |  |  |  |
| HHP | -0.783 | 0.450 |  |  |
| Duration of deafness_(Log2)_ | -0.253 | 0.097 | 0.115 |  |
| Age at activation_(Log2)_ | -0.903 | 0.261 | 0.575 | -0.058 |

## Supplementary Table 3.

Estimated correlations of fixed effects derived from the linear mixed-effects model investigating factors that influence Hearing Hours Percentage (HHP) in children with UHL+CI. For the categorical variable, onset of hearing loss, congenital was the reference variable. The comparison variable is in brackets.

|  | *(Intr)* | Duration of deafness_(Log2)_ | Time since activation_(Log2)_ | Age at Test | Onset of hearing loss [Progressive] |
| --- | --- | --- | --- | --- | --- |
| Duration of deafness_(Log2)_ | -0.407 |  |  |  |  |
| Time since activation_(Log2)_ | 0.363 | 0.078 |  |  |  |
| Age at Test | -0.573 | -0.288 | -0.274 |  |  |
| Onset of hearing loss [Progressive] | -0.327 | 0.115 | -0.006 | -0.066 |  |
| Onset of hearing loss [Sudden] | -0.118 | 0.347 | 0.125 | -0.415 | 0.293 |
